# Sulfidized Nanoscale Zerovalent Iron Supported by Oyster Powder for Efficient Removal of Cr (VI): Characterization, Performance, and Mechanisms

**DOI:** 10.3390/ma15113898

**Published:** 2022-05-30

**Authors:** Hao Hu, Donglin Zhao, Changnian Wu, Rong Xie

**Affiliations:** Key Laboratory of and Functional Molecule Design and Interface Process, Anhui Jianzhu University, Hefei 230601, China; huhao6031342@163.com (H.H.); wucnustc@126.com (C.W.); xr@ahjzu.edu.cn (R.X.)

**Keywords:** oyster shell, S-nZVI, adsorption, reduction, mechanism

## Abstract

In this study, sulfidized nanoscale zerovalent iron (S-nZVI) supported by oyster shell (OS) powder (S-nZVI@OS) was synthesized by controlling the initial S/Fe ratios (0.1–0.5) to explore the potential synergistic effects during the adsorption and reduction of Cr (VI). X-ray diffraction (XRD), transmission electron microscopy (TEM), and X-ray photoelectron spectroscopy (XPS) analyses showed that Fe (0) and FeS were well dispersed on the OS surface. Furthermore, the stability of S-nZVI@OS composite was higher than that of nZVI, which was proved by the material ageing experiment. The effects of different S/Fe molar ratios, time, temperature, the initial concentration of Cr (VI), and initial pH on the removal efficiency were also studied. The results indicated that with the increase of the S/Fe molar ratio, the removal capacity of Cr (VI) first increased rapidly and then decreased slowly. Batch experiments showed that an optimal S/Fe molar ratio of 0.2 offered a Cr (VI) removal capacity of about 164.7 mg/g at pH 3.5. The introduction of S can not only promote Cr (VI) reduction but also combine with Cr (III) by forming precipitate on S-nZVI@OS mainly as Cr_x_Fe_(1−x)_ OOH and Cr_2_S_3_. The adsorption thermodynamics and kinetics demonstrated that the Langmuir model and pseudo-second-order kinetics model can describe the adsorption isotherms and kinetics. These results suggest that S-nZVI@OS is an effective and safe material for removing Cr (VI) from aqueous solutions.

## 1. Introduction

Heavy metal–contaminated wastewater is a central problem in water pollution [1,2]. Controlling water pollution and protecting water resources have become essential goals globally. Chromium is one of the primary heavy metals causing groundwater pollution. With rapid industrial development, chromium is widely used in several industries, including electroplating, metallurgy, mechanical engineering, chemical engineering, and electronics [3]. Over the years, with the increase in industrialization worldwide, much chromium-containing wastewater discharge has polluted water supplies [4]. In general, chromium exists in nature in the form of Cr (VI), such as Cr_2_O_7_^2−^ and CrO_4_^2−^ [5,6,7,8], and Cr (VI) has been identified as a strong carcinogen [9,10]. The Ministry of Ecology and Environment of China has set the relevant limit values for Cr (VI) [11].

With rises in contamination, many tactics for removing Cr (VI) have been developed, including biological approaches [12], electrochemical oxidation methods, membrane filtering methods [13], and adsorption methods [14]. The adsorption method is regarded as the most promising way of eliminating Cr (VI) due to its high efficiency, cheap cost, and simplicity. The most commonly utilized substance for removing Cr (VI) is nanoscale zerovalent iron (nZVI) [15]. It has a high specific surface area, is highly reducible, and is low in toxicity [16]. However, particle agglomeration reduces the migration ability of nZVI in a porous environment. Moreover, nZVI exhibits low reaction efficiency with other chemicals in waste, which reduces pollutant removal efficiency [17]. The main methods of nZVI modification are emulsification, immobilization, vulcanization, and bimetallic particles. After several years of research, Han and Yang [18] found that nZVI could be modified by sodium thiosulate or sodium sulfide to increase its conductivity, inhibit its reaction with water, and extend its lifespan. Moreover, the nZVI-loaded metal-organic framework exhibited significant Cr (VI) removal capacity [19]. nZVI has been successfully loaded onto silica [20], activated carbon [21,22], zeolite [23], biochar [24], chitosan [25], metal [26], and other materials. However, nZVI materials still have some drawbacks. For example, the process of removing Cr (VI) requires highly acidic conditions, and the reaction produces large amounts of sludge containing heavy metals [27]. To further improve the reducing ability of nZVI, sulphur is usually added to the material to generate S-nZVI, with a stronger reducing ability to remove Cr (VI) [28].

Many sorbents, including biochar [29], clay [30], polymers [31,32] and graphene oxide [33], can be used as carriers. Oyster shell (OS) is a common marine debris in coastal cities. Its chemical constituents are mainly inorganic substances. OS is rich in calcium salts and has a porous surface. The shell comprises a cuticular layer, a prismatic layer and a pearl layer. The cuticular layer is highly resistant to corrosion, the prismatic layer has a foliated structure with a large number of 2~10 microns micropores, and the pearl layer is mainly calcite. Due to its special structure and calcium carbonate composition, OS can be decomposed into CaO and CO during high-temperature calcination, as well as CO_2_ gas. Moreover, its pore structure endows it with strong adsorption capacity, exchange capacity, and catalytic decomposition capacity. Therefore, it can absorb various pollutants in sewage and improve water quality. Through dynamic column experiments and static batch experiments, Gao et al. [34,35,36] studied the adsorption performance of OS powder towards cadmium and cobalt in an aqueous solution. The adsorbent exhibited better Cd^2+^ removal than that of Co^2+^ removal from single-component metal ion solutions. In addition, OS is self-alkaline, which facilitates Cr (III) precipitation. The treatment of domestic waste with OS powder is characterized by high efficiency, low consumption, and the absence of secondary pollution, demonstrating the potential applications of the powder.

It is reported that OS has not been used as a solid loading material for nZVI in the current literature. In this study, OS was used as the loading material to synthesize S-nZVI@OS, which was used to remove Cr (VI) from water. S-nZVI@OS was characterized via XRD, SEM, FTIR, and XPS. On Cr(VI) removal, the influence of pH, Cr(VI) starting concentration, and temperature was examined.

## 2. Materials and Methods

### 2.1. Chemical Reagent

Potassium dichromate (K_2_Cr_2_O_7_, 99.8%) was purchased from Tianjin Chemical Co., Ltd. Ferrous sulfate heptahydrate (FeSO_4_·7H_2_O) and phosphoric acid were purchased from McLaines Biochemical Technology Co., Ltd. (Shanghai, China), and sodium sulfide monohydrate (Na_2_S·9H_2_O) was bought from Fuchen Reagent Co., Ltd. Sodium borohydride (NaBH_4_) was purchased from Damao Chemical Reagent Factory (Tianjin, China). Oyster powder was purchased from Guangxi. The original solution containing potassium dichromate (500 mg/L) was prepared by drying 0.2829 g of K_2_Cr_2_O_7_ pellets at high temperature for a period of time and then dissolving them in deionized water.

### 2.2. The Preparation of S-nZVI@OS

**Figure 1:**
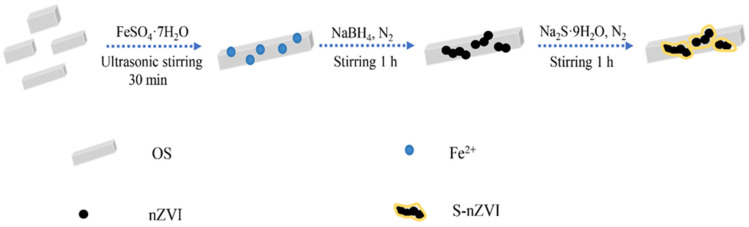
Schematic diagram of S-nZVI@OS synthesis.

Composite diagram of S-nZVI@OS is shown in Figure 1. The S-nZVI@OS was prepared via the following processes: (1) To maximize contact between oyster powder and iron, 1.1 g of oyster powder was dispersed in 5.5 g of 100 mL of FeSO_4_·7H_2_O solution under ultrasonic conditions and stirred for 30 min. (2) The mixture was stirred with a mechanical stirrer under N_2_ conditions for 1 h, and then borohydride (3 g, 50 mL) was slowly added with a constant-pressure separator to reduce the iron ions in the solution. (3) Na_2_S·9H_2_O was deposited into the mixture. The introduction of sulfide turns the system into FeS, and an FeS layer is formed on the nZVI surface when the system contains a large amount of Fe^2+^. The resulting material was cleaned with anaerobic water and anhydrous ethanol many times before being vacuum dried and stored in a vacuum glove box until it was needed again.

### 2.3. Characterization and Analysis Methods

The composite crystal structures were characterized using X-ray diffraction (XRD, Bruker D8 Advance, Germany) scanned in the range of 10–80° (2θ). The morphology of the product was studied by transmission electron microscope (Jeol Jem-2100, TEM) images and scanning electron microscope (Regulus8100, SEM) images. An electronic spectrometer (XPS, PHI-5300, UK) for analyzing the state of surface elements using X-ray photographs of chemical elements. In addition, a zeta potential meter (Malvern Zetasizer Nano ZS 90, UK) and a vibrating sample magnetometer (VSM LakeShore 7400-S, USA) were used to analyse the surface charge and magnetic properties of the material. Total Cr was determined by (ICP-OES) (Optima 8000, USA) and Cr (VI) concentration was determined by a spectrophotometric UV spectrophotometer (T6 New Century, China).

### 2.4. Removal Process

In 50 mL test tubes (25 °C), experiments on the adsorption of Cr (VI) by the new material S-nZVI@OS were carried out. A certain concentration of S-nZVI@OS was put into a 20 mg/L solution of Cr (VI) and then the pH was adjusted to 3.5 using various concentrations of HCl or NaOH. Conical flasks were used to study the removed material by shaking them in a temperature-controlled water bath shaker at 180 rpm. At the end of the specified experimental time, the adsorbent material is separated from the water phase using a magnet. All of the experiments were conducted three times.

Equation (1) was used to analyze Cr (VI) removal efficiency (RE), while Equation (2) was used to estimate Cr (VI) removal capacity (RC):

(1) $$\mathrm{RE}\left(\%\right)=\frac{\left(C_{0}-C_{t}\right)}{C_{0}}\times 100\%$$

(2) $$\mathrm{RC}=\frac{\left(C_{0}-C_{t}\right)\cdot V}{m}$$

where *C*_0_ is the initial Cr (VI) concentration (mg/L) and *C_t_* is the equilibrium Cr (VI) concentration (mg/L); *m* is the adsorbent mass (g); and *V* is the total of the aqueous systems in the reaction system (mL).

Quantitative relationships between Cr (III) concentrations, Cr (VI) concentrations, and total Cr concentrations:

(3) $$C_{Cr3+}+C_{Cr6+}=C_{TCr}$$

where *C_TCr_*, *C_Cr_*_6+_, and *C_Cr_*_3+_ are the *TCr*, Cr (VI), and Cr (III) concentrations in the reaction system (mg/L), respectively.

### 2.5. Kinetic Study

Kinetic experiments were performed using a three-necked flask. The experiments were performed at the same temperature under mechanical stirring. The Cr (VI) content was determined according to the amount of supernatant absorbed within a specified time.

The kinetic data were analyzed using the Langmuir–Hinshelwood first-order kinetic model Equation (4) and a pseudo-second-order kinetic model Equation (5) [37,38].

(4) $$\ln\left(C_{t}/C_{0}\right)=-k_{obs}t+c$$

(5) $$\;\frac{t}{q_{t}}=\frac{1}{k_{2}q_{e}^{2}}+\frac{t}{q_{e}}$$

where *q_e_* and *q_t_* are the Cr (VI) removal capacities at equilibrium and time *t* (mg/g), respectively; *k_obs_* (min^−1^) is the Langmuir–Hinshelwood first-order kinetic model’s rate constant; *c* is a constant; and *k*_2_ (g∙min/mg) is the pseudo-second-order rate constant.

### 2.6. Isotherms and Thermodynamics

The Langmuir Equation (6) and Freundlich isotherms Equation (7) are common adsorption models.

(6) $$\mathrm{Langmuir}:\;\;\frac{C_{e}}{q_{e}}=\frac{1}{q_{m}b}+\frac{C_{e}}{q_{m}}$$

(7) $$\mathrm{Freundlich}:\;\ln q_{e}=\ln k+\frac{1}{n}\ln C_{e}$$

The adsorbent concentration in solution at adsorption equilibrium is *C_e_* (mg/L), *q_e_* (mg/g) is the removal capacity of the material for Cr (VI), *q_m_* is the maximum removal capacity of the material for Cr (VI) (mg/g), and *b* (L/mg) is the Langmuir model constant associated with the adsorbent to the adsorbate. The Freundlich constant is *k*, and the adsorption strength is *n* (L/mg).

The thermodynamic parameters are determined by the following relations:

(8) $$\Delta G^{0}=-RT\ln K$$

(9) $$\ln K=\frac{\Delta S}{R}-\frac{\Delta H}{RT}$$

The free energy Δ*G*^0^ (kJ/mol), the enthalpy changes Δ*H*^0^ (kJ/mol), and the entropy change Δ*S*^0^ (J/mol/K) were all calculated. *R* is the ideal gas constant (8.314 J/(mol·K)) and *T* is the temperature in Kelvin (K). ln*K* is obtained by plotting ln*K*_d_ as a function of *C_e_*, with *C_e_* extrapolated to zero. Δ*S*^0^ and Δ*H*^0^ are the intercept and slope of a linear plot between ln*K* and 1/*T*, respectively.

## 3. Results and Discussion

### 3.1. Characterization

**Figure 2:**
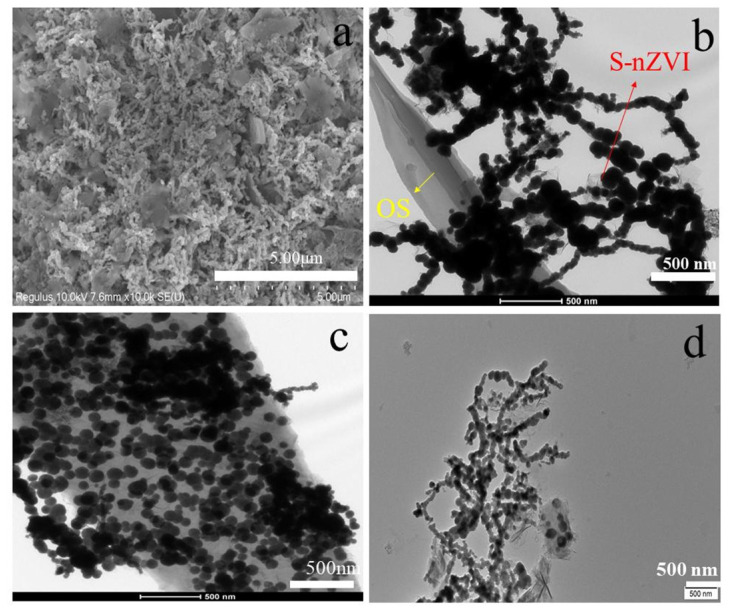
(**a**) SEM image of S-nZVI@OS; (**b**,**c**) TEM images of S-nZVI@OS; (**d**) TEM image of S-nZVI@OS after reaction.

Figure 2 shows SEM and TEM images of the material before and after reaction. SEM image of S-nZVI@OS is shown in Figure 2a. TEM images (Figure 2b,c) show that the dendritic S-nZVI is uniformly dispersed on the surface of the OS carrier and are more dispersed compared to nZVI, so the material has better spatial stability. Figure 2d shows essentially no change in the structure of the material compared to TEM images (Figure 2b,c) before reaction, which is further evidence of the stability of the material.

**Figure 3:**
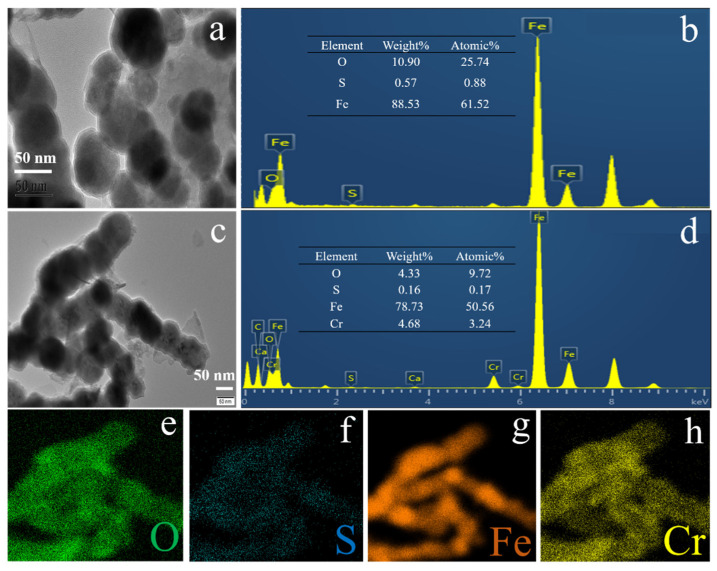
(**a**,**b**) TEM image of S-nZVI@OS and corresponding EDS images before reaction; (**c**–**h**) TEM image of S-nZVI@OS and corresponding EDS mapping images after reaction.

The elemental composition of S-nZVI@OS (Figure 3b) illustrates the presence of Fe, O and S elements in S-nZVI@OS, demonstrating the formation of Fe oxides and the presence of S after sulphide modification. Figure 3c–h show the HRTEM image of S-nZVI@OS and the corresponding EDS mapping after reaction. In comparison to Figure 3b, the mass concentration of S before reaction reduced from 0.57% to 0.16% after reaction, whereas the mass content of Fe reduced from 88.53% to 78.73%. This indicates that oxidation of S and Fe occurs during the removal process, resulting in the formation of SO_4_^2−^ and oxygenated iron compounds.

**Figure 4:**
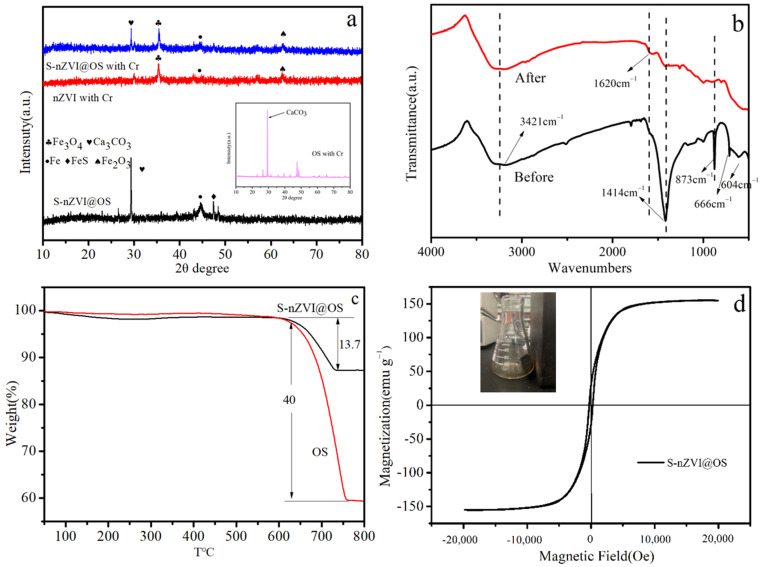
(**a**) XRD patterns of different composite before and after removal Cr (VI) (inset figure is the XRD pattern of OS after reaction with Cr (VI)); (**b**) FTIR spectra of S-nZVI@OS before and after reaction; (**c**) TG thermogram of OS and S-nZVI@OS; (**d**) magnetization curve of S-nZVI@OS.

Figure 4a describes powder XRD patterns of different composite before and after removal Cr (VI). The peak at 2θ of 29.4° is CaCO_3_ (PDF#05-0586), corresponding to the (104) plane of the index. In the S-nZVI@OS composite, the crystalline metals Fe (PDF#99-0064) and FeS (PDF#23-1123) were present due to the diffraction peaks at 44.67° and 47.46°, corresponding to the index (110) and (220) planes, respectively. After reaction with Cr (VI), new characteristic peaks appeared at 35.62° and 62.59° indexed as (3 1 1) and (2 1 4) planes corresponding to the characteristic peaks of Fe_3_O_4_ (PDF#88-0315) and Fe_2_O_3_ (PDF#72-0469). The presence of iron oxides after reaction indicates that the iron was oxidized during the removal process. The XRD pattern of OS after reaction with Cr (VI) shows there is no characteristic peak of new substance, which indicates that OS mainly participates in the adsorption.

The FTIR spectra of the composite material before and after the reaction are shown in Figure 4b. Prior to the reaction, peak locations are 3421 cm^−1^, 1620 cm^−1^, 1414 cm^−1^, and 873 cm^−1^, corresponding to stretching vibrations related to hydroxyl (-OH), C=O bonds, carboxyl (-COOH), and C-O bonds, respectively. The disappearance of absorption vibration absorption peaks for carboxyl and C-O bonds following reaction indicates a reduction reaction during Cr (VI) removal. In addition, the spectrum of the S-nZVI@OS composite exhibits absorption peaks at 666 and 604 cm^−1^, corresponding to the formation of Fe-O and Fe-S bonds during the preparation of the composite [39]. In contrast, the vibrational absorption peak here disappears after the reaction, indicating that the oxidation of Fe and S occurred during the removal process.

Figure 4c shows the results of the thermogravimetric analysis of OS and S-nZVI@OS. Both materials have mass loss at 630 °C, and the weight loss of OS is 40%, which is due to the decomposition of CaCO_3_ contained in OS. S-nZVI@OS loses 13.7% of its weight owing to the decomposition of OS, which indicates that S-nZVI loaded by OS has good thermal stability. Figure 4d is the magnetization curve of S-nZVI@OS. It can be clearly seen that the magnetization value of S-nZVI@OS is high (97 emu/g), which indicates that the adsorbent can be recycled using magnetism well after the reaction is completed.

### 3.2. Adsorption Kinetics for S-nZVI@OS with Different S/Fe Ratio

**Figure 5:**
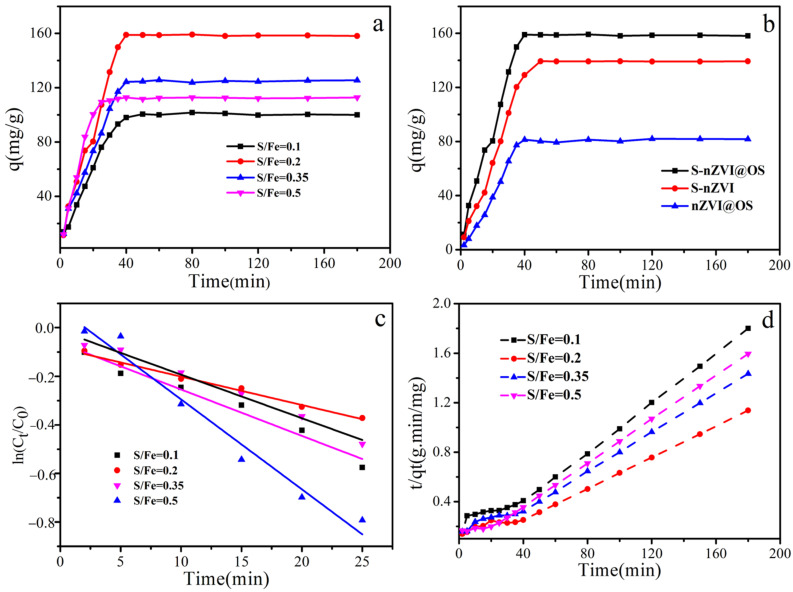
(**a**) The adsorption kinetics of Cr (VI) with different S/Fe ratios; (**b**) the adsorption kinetics of S-nZVI, nZVI@OS and S-nZVI@OS; (**c**) Liner polts of lnC_t_/C_0_ versus T; (**d**) pseudo-second-order dynamics diagram.

Figure 5a depicts the kinetics of Cr (VI) elimination for materials with various S/Fe ratios. The Cr (VI) removal rate was high for the first 30 min and then levelled off with time. The equilibrium adsorption capacities were 100, 158, 125, and 112 mg/g for S/Fe ratios of 0.1, 0.2, 0.35, and 0.5, respectively. The results reveal that as the S/Fe ratio increases, the Cr (VI) removal capability increases at first, then falls. A too-high S/Fe ratio may lead to the formation of less active FeS_n_, which reduces the reduction capacity of Fe (0) and the activity of the particles [40]. Therefore, the S/Fe ratio affects the effectiveness of the material in removing Cr (VI).

Figure 5b illustrates the Cr (VI) adsorption kinetics of S-nZVI@OS, S-nZVI and nZVI@OS. Adsorption was rapid in the first 40 min and then entered the adsorption equilibrium phase. The maximum adsorption capacities of S-nZVI@OS and S-nZVI were 158 mg/g and 138 mg/g, respectively. In a previous study, biochar was a commonly used loading material for studying nZVI, nZVI/biochar (0.1 g/L) exhibited a removal rate of approximately 30% toward 10 mg/L Cr (VI) [24]. nZVI@OS had a removal rate of about 40% under the same settings. Thus, nZVI@OS has a much greater Cr (VI) removal efficiency than that of biochar-supported nZVI. 

Figure 5c,d depict the Langmuir–Hinshelwood first-order kinetic model and the pseudo-second-order kinetic model, respectively. Table 1 shows the fitted parameters and correlation coefficients. By comparing the findings of the correlation coefficients (R^2^), it is shown that the pseudo-second order kinetic model can better describe the process of Cr (VI) elimination. The results reveal that rather than interfacial resistance, the transfer of valence electrons determines Cr (VI) removal [41,42].

### 3.3. Effect of Initial Solution pH and Ionic Strength

**Figure 6:**
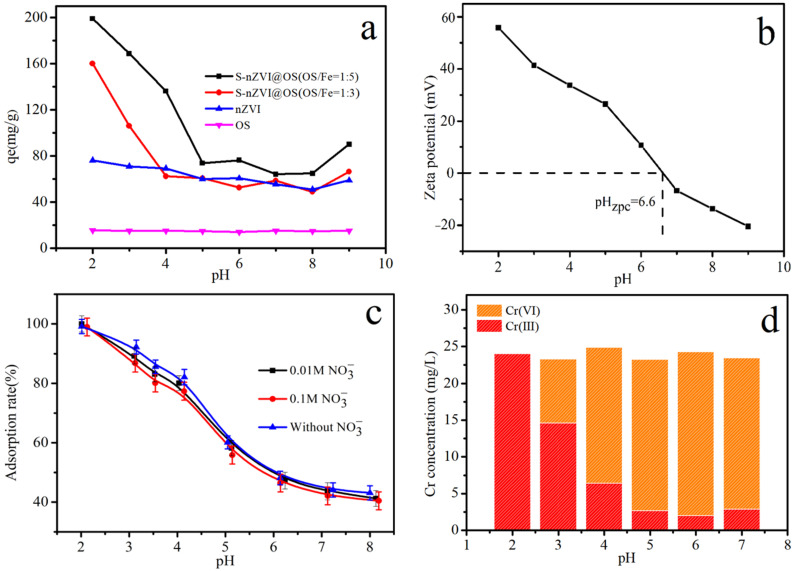
(**a**) The adsorption capacity of OS, nZVI and S-nZVI@OS for Cr (VI) under different initial pH; (**b**) Zeta potential of S-nZVI@OS; (**c**) Effect of ionic strength at different initial pH on Cr (VI) removal; (**d**) residual concentration of total Cr, Cr (VI) and Cr (III) after reaction in solution.

Figure 6a shows the Cr (VI) removal capacity of OS, nZVI, S-nZVI@OS (OS:Fe = 1:3) and S-nVI@OS (OS:Fe = 1:5) at the same initial concentration and different pH values. At an initial pH of 2–3.5 in aqueous solution, the removal efficiency of Cr (VI) (20 mg/L) by S-nZVI@OS (0.1 g/L, OS:Fe = 1:5) exceeded 84% in aqueous solutions with an initial pH of 2–3.5, while the removal efficiency by S-nZVI@OS (OS:Fe = 1:3) was 40–80%. Therefore, as the ratio of OS to Fe increased, S-nZVI@OS was detrimental to the removal of Cr (VI). This indicates that OS plays a role in the removal process by adsorption and that an increase in the amount of OS decreases the reduction of S-nZVI. When the pH value is 3.5–9, the removal rate of Cr (VI) by S-nZVI@OS decreases and the removal rate is lower than 32%. Therefore, S-nZVI@OS had greater Cr (VI) removal capacity under acidic conditions. A previous study reported that Cr (VI) removal by nZVI-based materials is an acid-mediated process [43]. Figure 6b show the zeta potential of S-nZVI@OS at different initial pH was investigated, and the results showed a value of 6.6 for pH_ZPC_. It can be shown that the S-nZVI@OS complex is stable in an aqueous solution under acidic conditions [24]. Acidic conditions favor Cr (VI) removal since H^+^ accelerates iron corrosion and reduces the material’s surface passivation. 

Considering the actual situation of Cr (VI) removal, the ionic strength affects the removal effect. Therefore, the effect of different NO_3_^−^ concentrations on the removal of Cr (VI) was investigated in the pH range 2–8. As shown in Figure 6c, the removal of Cr (VI) by S-nZVI@OS was clearly influenced by pH and the ionic strength (0.01 M NO_3_^−^, 0.1 M NO_3_^−^) had no obvious effect, which confirmed that the removal process belonged to the inner sphere surface complexation. Inner-sphere surface complexation implies that Cr (VI) is adsorbed by functional groups on the surface of the adsorbent and then immobilised in the interior of the hydration sheath [44]. Figure 6d depicts the changes in total Cr, Cr (VI), and Cr (III) concentrations. The concentration of Cr (VI) drops in an acidic environment while Cr (III) increases, showing that most of the Cr (VI) is transformed to Cr (III).

### 3.4. Adsorption Isotherms and Thermodynamics

**Figure 7:**
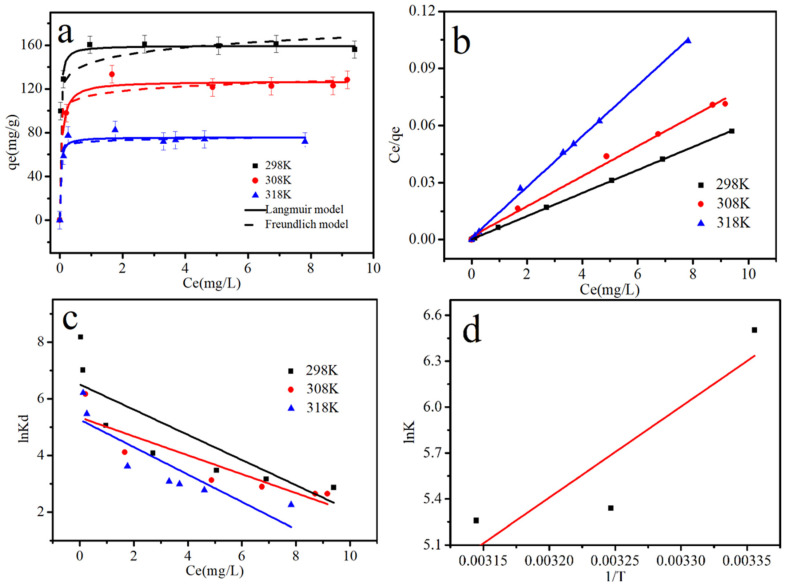
(**a**) Adsorption isotherms for Cr (VI) on S-nZVI@OS at 298, 308 and 318 K; (**b**) Langmuir plot; (**c**) linear plots of lnK_d_ versus *C_e_*; (**d**) linearized Arrhenius plot of ln*K* as a function of 1/*T*.

Figure 7a shows the isotherms for Cr (VI) adsorption at 298, 308, and 318 K on S-nZVI@OS. The Cr (VI) adsorption capacity declined with increasing temperature, from 164.745 mg/g to 75.075 mg/g, indicating an exothermic adsorption mechanism. Langmuir Equation (6) and Freundlich Equation (7) models were used to investigate the adsorption equilibrium of S-nZVI@OS. The correlation coefficients are presented in Table 2. Table 3 lists the comparison of Cr (VI) removal by S-nZVI@OS and other different modified materials [45,46,47,48,49,50,51,52]. The adsorption performance of OS and the excellent reduction ability of S-nZVI make S-nZVI@OS a potential and promising material to capture Cr (VI) from an aqueous solution.

According to the fitted results, the Langmuir model better described the experimental data (Table 4). The free energy Δ*G*^0^ (kJ/mol), enthalpy change Δ*H*^0^ (kJ/mol), and entropy change Δ*S*^0^ (J/mol/K) were calculated using Equations (8) and (9) to investigate the exothermic features of the adsorption process. Lower temperatures resulted in significant and negative Δ*G*^0^ values, indicating that the process was spontaneous and that lower temperatures were favorable. The calculation of Δ*H*^0^ < 0 and Δ*S*^0^ < 0 shows that the adsorption process is spontaneous exothermic.

### 3.5. Effect of Material Ageing on Removal Efficiency

**Figure 8:**
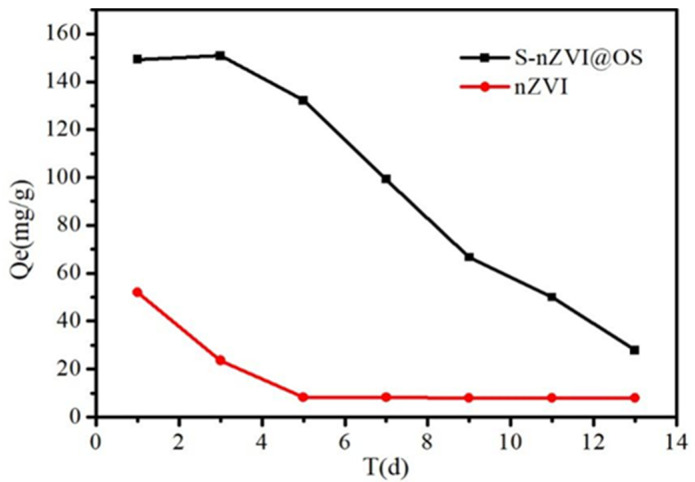
Changes in adsorption capacity of S-nZVI@OS and nZVI@OS over time.

Agglomeration and anodization of the nZVI layer occur with age, reducing the surface activity of nZVI and affecting Cr (VI) removal [53]. In the current study, the S-nZVI@OS composite maintained a high Cr (VI) removal rate after 14 days (Figure 8). FeS_X_ possibly reduced the magnetic attraction between nZVI particles, thereby inhibiting particle agglomeration. Moreover, the sulfide will inhibit the side reaction of the material with water, which helps to preserve the high activity of the material. Comparing the TEM images before and after the reaction, the structure of the material is almost unchanged before and after the reaction. This further demonstrates the stability of the new composite S-nZVI@OS in terms of Cr (VI) removal [54].

## 4. Mechanism Analysis

**Figure 9:**
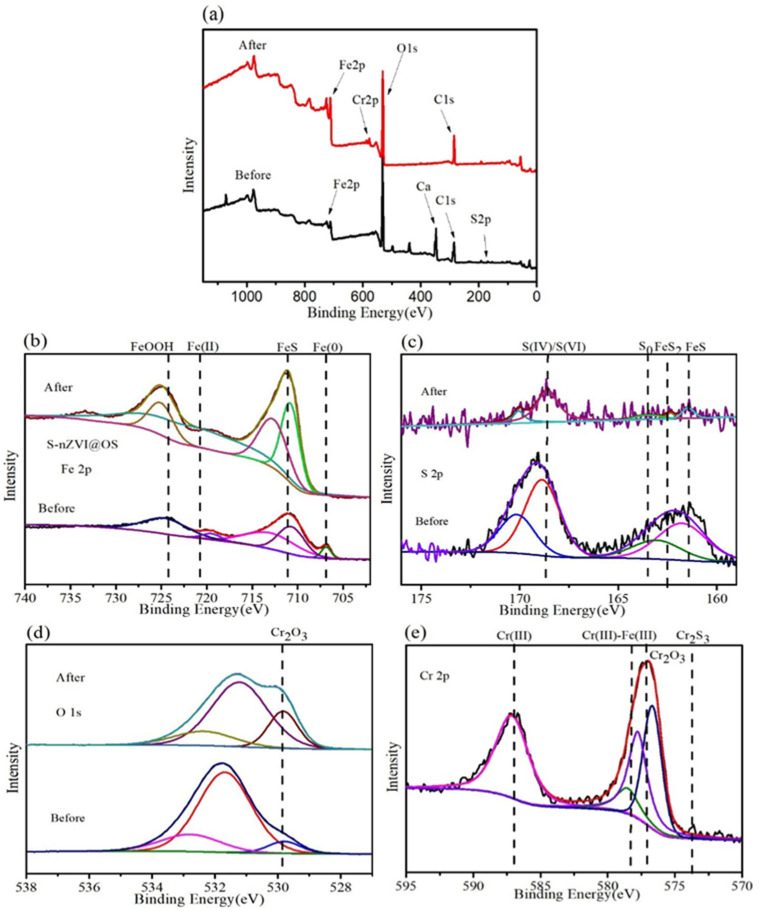
(**a**) XPS survey of S-nZVI@OS before and after reaction with Cr (VI); (**b**) Fe2p spectra before and after reaction; (**c**) S2p spectra before and after reaction; (**d**) O1s spectra before and after reaction; (**e**) Cr2p spectrum.

The Fe 2p3/2 XPS spectra of S-nZVI@OS are shown in Figure 9b. Fe^0^ and Fe (III) are represented by peaks with binding energies of 707.5 eV and 724.3 eV, respectively, whereas Fe^2+^ is represented by peaks with binding energies of 711.6 eV and 721.9 eV [53]. The spectrum of the fresh S-nZVI@OS (i.e., before reaction) exhibited the Fe^0^ characteristic peak. The peaks of Fe^0^ and Fe^2+^ move to high binding energy peak positions in the spectrum of S-nZVI@OS (i.e., after reaction). The peaks at 724.3 eV and 725 eV represent the production of FeOOH and FeCr_2_O_4_. This suggests that the process occurs with the oxidation of Fe^0^ and the precipitation formation of FeOOH.

The S 2p spectrum (Figure 9c) prior to the reaction shows peaks at 161.9, 164.3, 162.5, and 169 eV, corresponding to S (-II), S (0), FeS_2_, and S (IV), respectively [55]. From the fractional spectrum of S 2p before and after the reaction (Figure 9c), S is mainly present as S (-II), FeS_2_, S (IV), and S (VI). Thus, S (-II) and S (0) were oxidized to S (IV)/S (VI) by Cr (VI) under acidic conditions (pH = 3.5). This indicates that Cr removal was also based on a reduction reaction.

The O 1s spectrum of S-nZVI@OS (Figure 9d) exhibited three peaks. Three peaks appear at 530.1, 531.6 and 533.2 eV, which correspond to an oxide (O^2−^), a surface hydroxyl group (-OH) and adsorbed water (H_2_O), respectively. Iron oxide and hydroxide compounds existed on the S-nZVI@OS surface before the reaction. After the reaction, the intensity of the oxide (O^2−^) peak increased, and it can be judged that the main product was Cr_2_O_3_.

Figure 9e shows the Cr 2p XPS spectrum. Three peaks with binding affinity of 578.7, 587, and 574 eV occur, with 578.7 eV corresponding to Cr (III)-Fe (III) and 587 and 574 eV belonging to Cr (III) and Cr_2_S_3_, respectively [56]. The Cr (VI) binding energy weakens after the reaction, whereas the Cr (III)-Fe (III) and Cr (III) binding energies become significantly stronger. These modifications suggest that Cr (VI) was reduced and forms Cr (III)-Fe (III) complexes on the adsorbent surface.

**Figure 10:**
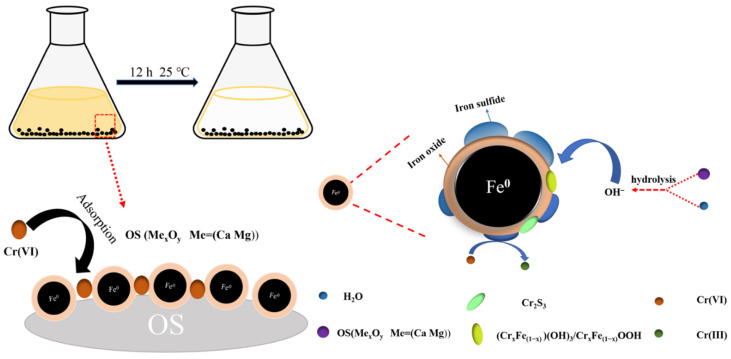
Schematic representation for mechanistic pathway of Cr (VI) reduction by S-nZVI@OS.

The removal mechanism is shown in Figure 10. The elimination of Cr (VI) can be separated into three steps, according to the analysis. Firstly, Cr (VI) can be trapped just on material surface due to its substantial positive charge and porosity. Secondly, electrons are transferred from Fe^0^, Fe^2+^, S^2−^, and S to Cr (VI), which is reduced to Cr (III). Fe^0^, Fe^2+^ and S are oxidized to Fe_2_O_3_, Fe_3_O_4_, and SO_4_^2−^. Finally, the metal oxides contained in OS will generate a large number of metal cations (Ca^2+^) under acidic conditions. Cations in the aqueous solution are hydrolyzed to generate a large amount of OH^-^, thus generating (Cr_x_Fe_(1−x)_) (OH)_3_ and Cr_x_Fe_(1−x)_ OOH precipitates. A considerable amount of H^+^ in the solution is consumed during the electron transfer process. Therefore, the solution pH increased from 3.5 to 5.8. In summary, the OS played an essential role in not only the dispersion, maintenance, and minimization of S-nZVI particles but also the adsorption and precipitation processes. The specific reaction process was presented as Equations (10)–(15) [56,57]. The combined action of Fe (0), S (0), Fe^2+^, and S^2−^ removed Cr (VI). The reaction requires fewer hydrogen ions than those associated with other materials, which makes S-nZVI@OS advantageous. Furthermore, oxidized S, Fe (III), and Cr (III) were formed as sulfates and precipitates, reducing the material’s passivation effect.

(10) $${\mathrm{Cr}}_{2}O_{7}^{2-}+3{\mathrm{Fe}}^{0}+14H^{+}\to 2{\mathrm{Cr}}^{3+}+3{\mathrm{Fe}}^{2+}+7H_{2}O$$

(11) $$\left(1-x\right){\mathrm{Fe}}^{3+}+{\mathrm{xCr}}^{3+}+3H_{2}O\to \left({\mathrm{Cr}}_{x}{\mathrm{Fe}}_{\left(1-x\right)}\right){\left(\mathrm{OH}\right)}_{3}\left(s\right)+3H^{+}$$

(12) $$\left(1-x\right){\mathrm{Fe}}^{3+}+{\mathrm{xCr}}^{3+}+2H_{2}O\to {\mathrm{Cr}}_{x}{\mathrm{Fe}}_{\left(1-x\right)}\mathrm{OOH}\left(s\right)+3H^{+}$$

(13) $${\mathrm{HCrO}}_{4}^{-}+3{\mathrm{Fe}}^{2+}+7H^{+}\to {\mathrm{Cr}}^{3+}+3{\mathrm{Fe}}^{3+}+4H_{2}O$$

(14) $${\mathrm{Cr}}_{2}O_{7}^{-}+S^{0}+5H^{+}\to 2{\mathrm{Cr}}^{3+}+{\mathrm{SO}}_{4}^{2-}+3H_{2}O$$

(15) $$2{\mathrm{Cr}}^{3+}+3S^{2-}\to {\mathrm{Cr}}_{2}S_{3}\left(s\right)$$

## 5. Conclusions

S-nZVI@OS, a ternary composite prepared in two steps, successfully removed Cr (VI) from water. TEM and SEM results demonstrated that the particles were uniformly dispersed, with minimal agglomeration. The Langmuir isotherm model well describes the experimental data. The Langmuir–Hinshelwood first-order kinetic model and a pseudo-second-order kinetic model agreed well with the kinetics of Cr (VI) removal. Under acidic conditions (low solution pH), the new ternary composite S-nZVI@OS exhibits a powerful removal of Cr (VI). In addition, S-nZVI@OS has shown excellent performance in terms of stability. The highest removal rate was achieved at a solution pH of 3.5 with an S-nZVI@OS dosage of 0.1 g/L. Adsorption isotherms were obtained at 298, 308 and 318 K. The free energies Δ*G*^0^ (kJ/mol) were obtained as −15.695, −14.562 and −13.429 kJ/mol, respectively, the enthalpy changes Δ*H*^0^ (kJ/mol) and entropy change Δ*S*^0^ (J/mol/K) were −49.457 kJ/mol and −113.294 J/mol/K. The results showed that the lower temperature was favorable for the reaction, and the corresponding adsorption capacity of S-nZVI@OS was 164.7 mg/g at 298 K. XPS analysis shows that the process of S-nZVI@OS in the removal of Cr (VI) is an integrated adsorption and reduction process. Self-alkaline loading material OS not only has excellent adsorption performance, but also contributes to the formation of (Cr_x_Fe_(1−x)_) (OH)_3_ and Cr_x_Fe_(1−x)_ OOH precipitates through hydrolysis. To summarize, OS-supported S-nZVI@OS is a low-cost, effective, and ecologically friendly Cr (VI) removal material.

## Figures and Tables

**Table 1:** Kinetic parameters of Langmuir-Hinshelwood first-order kinetics model and pseudo-second-order kinetics model of S-nZVI@OS with different S/Fe ratios.

S/Fe Ratio	Langmuir-Hinshelwood First-Order Kinetics Model			Pseudo-Second Order Model	
	K_obs_ (min^−1^)	R^2^	k_2_ (g/mg min)	Q_e_ (mg/g)	R^2^
0.100	0.018	0.990	0.647 × 10^−3^	112.996	0.985
0.200	0.012	0.990	0.352 × 10^−3^	178.473	0.982
0.350	0.019	0.975	0.514 × 10^−3^	139.538	0.988
0.500	0.037	0.974	1.240 × 10^−3^	119.764	0.992

**Table 2:** Isotherm parameters of S-nZVI@OS removal of Cr (VI).

T(K)	Langmuir Model			Freundlich Model		
	Q_max_ (mg/g)	b (L/mg)	R^2^	k (mg·g^−1^)	*n*	R^2^
298	164.745	21.328	0.999	143.314	12.402	0.908
308	126.582	4.463	0.994	104.921	14.760	0.760
318	75.075	11.684	0.998	66.567	17.077	0.925

**Table 3:** Comparison of Cr (VI) removal by various modified nZVI materials.

Adsorbents	pH	Q_max_ (mg/g)	References
S-nZVI@OS	3.5	164.7	This work
Biochar-CMC-nZVI	5.6	112.5	[45]
nZVI/Cu	5.0	18.8	[46]
nZVI@HCl-BC	5.0	17.8	[47]
TP-nZVI-OB	2.0	95.5	[48]
SBC-nZVI	3.0	84.4	[49]
nGO-nZVI	7.0	21.7	[50]
Sepiolite/nZVI	6.0	43.9	[51]
CS-nZVI	4.0	101.8	[52]

**Table 4:** Thermodynamic parameters of Cr (VI) removal by S-nZVI@OS at different temperatures (298, 308, 318 K).

T (K)	Δ*G*^0^ (kJ/mol)	Δ*H*^0^ (kJ/mol)	Δ*S*^0^ (J/mol/K)
298	−15.695		
308	−14.562	−49.457	−113.294
318	−13.429		

## Data Availability

The data presented in this study are available on request from the corresponding author.
